# *Bacillus*-based biocontrol beyond chemical control in central Africa: the challenge of turning myth into reality

**DOI:** 10.3389/fpls.2024.1349357

**Published:** 2024-02-06

**Authors:** Gaspard Nihorimbere, Virginie Korangi Alleluya, François Nimbeshaho, Venant Nihorimbere, Anne Legrève, Marc Ongena

**Affiliations:** ^1^ Phytopathology- Applied Microbiology, Earth, and Life Institute, UCLouvain, Louvain-la-neuve, Belgium; ^2^ Unité de défense des végétaux, Institut des Sciences Agronomiques du Burundi, Bujumbura, Burundi; ^3^ Microbial Processes and Interactions, TERRA Teaching and Research Center, Gembloux Agro-Bio Tech, University of Liège, Gembloux, Belgium; ^4^ Chemical and Agricultural Industries, Faculty of Agricultural Sciences, University of Kinshasa, Kinshasa, Democratic Republic of Congo; ^5^ Laboratoire de Nutrition-Phytochimie, d’Ecologie et d’Environnement Appliquée, Centre Universitaire de Recherche et de Pédagogie Appliquées aux Sciences, Institut de Pédagogie Appliquée, Université du Burundi, Bujumbura, Burundi; ^6^ Département des Sciences et Technologie des Aliments, Faculté de Bio-Ingénierie, Université du Burundi, Bujumbura, Burundi

**Keywords:** Burundi, Rwanda, DRC, diseases and pests, pesticide, IPM, *Bacillus* spp., biocontrol

## Abstract

Agricultural productivity in the Great Lakes Countries of Central Africa, including Burundi, Rwanda, and the Democratic Republic of Congo, is affected by a wide range of diseases and pests which are mainly controlled by chemical pesticides. However, more than 30% of the pesticides used in the region are banned in European Union due to their high toxicity. Globally available safe and eco-friendly biological alternatives to chemicals are virtually non-existent in the region. *Bacillus* PGPR-based biocontrol products are the most dominant in the market and have proven their efficacy in controlling major plant diseases reported in the region. With this review, we present the current situation of disease and pest management and urge the need to utilize *Bacillus*-based control as a possible sustainable alternative to chemical pesticides. A repertoire of strains from the *Bacillus subtilis* group that have shown great potential to antagonize local pathogens is provided, and efforts to promote their use, as well as the search for indigenous and more adapted *Bacillus* strains to local agro-ecological conditions, should be undertaken to make sustainable agriculture a reality in the region.

## 1 Introduction

The importance of agriculture for the development of societies has been demonstrated for several centuries and it employs about 43% of the world’s working population (Roser, 2023). In the Great Lakes Countries of Central Africa (GLCCA), referred to in this report as Burundi, Rwanda, and the Democratic Republic of Congo (DRC), agriculture is the main activity employing more than 94%, 46% and 60% of the working population, while contributing to 40%, 30% and 36% of gross domestic product (GDP) in Burundi, Rwanda, and DRC respectively (PND, 2018; Lokuruka, 2020; FAAPA, 2021). However, this sector faces several constraints including low soil fertility and high incidence of diseases and pests leading to food insecurity (Bjornlund et al., 2020). For example, up to 52%, 43% and 38% of children under five in Burundi, DRC and Rwanda respectively are malnourished (Lokuruka, 2020; WFP, 2021; FAO, 2023). In the GLCCA, plant diseases and pests are generally controlled by chemical pesticides, especially those affecting cash crops such as coffee, cotton, tomatoes, potatoes, vegetables, and fruits (Niyongere et al., 2015; Muliele et al., 2018; Okonya et al., 2019a). However, some phytopathogens are inefficiently managed by traditional practices, such as the use of plant extracts, while others are not controlled (Rutikanga, 2015; MINAGRI-Burundi, 2018; Korangi Alleluya et al., 2021).

Biocontrol products are the most promoted tools within the Integrated Pest Management (IPM) framework, as they are generally recognized as safe (GRAS) compared to chemical pesticides (Raveau et al., 2020). Biocontrol products include biochemicals (semiochemicals, plant extracts, plant growth regulators and organic acids), macroorganisms (insects, mites, and nematodes) and microorganisms (beneficial bacteria, fungi, protozoa, viruses, yeasts) and their derivatives (cyclic lipopeptides, enzymes, chitosan oligopolysaccharides, etc…) (DunhamTrimmer, 2023). Among microorganisms, plant growth-promoting rhizobacteria (PGPR), including species of the *Streptomyces*, *Paenibacillus*, *Pseudomonas* and *Bacillus* genera, are the most commercially exploited for crop bioprotection, notably due to their efficient production of an arsenal of bioactive secondary metabolites (BSMs). These bacteria protect plants through direct antibiosis, competition with other pathogenic microbes for space and nutrients, and via their potential to induce plant systemic resistance (ISR) (Beneduzi et al., 2012; Wang et al., 2021; Dimkić et al., 2022). *Bacillus*-based products dominate the biocontrol market compared to their PGPR counterparts (DunhamTrimmer, 2023; Helepciuc and Todor, 2023) due to their ability to form resistant endospores, allowing stable formulations and ensuring long-term survival in the environment, especially in the current climate change context (Radhakrishnan et al., 2017; Miljaković et al., 2020). In this review, we provide a detailed topography of plant health and crop protection in the GLCCA, address constraints within the sector, and propose promising sustainable solutions. An overview of biological alternatives to chemical pesticides is discussed, with a focus on *Bacillus* PGPR for potential integration as biocontrol ingredients into local agricultural systems.

## 2 Diversity of phytopathogens and their control

We performed a comprehensive survey of the major diseases and causal agents affecting crop production in the GLCCA region by compiling data from published studies (articles and books) with those obtained from governmental and non-governmental agencies in the form of reports, newspapers, or online databases. Either in the field or in storage, agricultural crops are mainly affected by microbial pathogens consisting of fungi, oomycetes, bacteria, and viruses (**Table 1**). The most important fungal diseases encompass late blight of potato and tomato caused by *Phytophthora infestans*, angular leaf spot of bean caused by *Pseudocercospora griseola*, early blight of tomato caused by *Alternaria solani*, stem rot caused by *Sclerotium rolfsii*, late and early leaf spot caused by *Nothopassalora personata* and *Cercospora arachidicola* on peanut. These pathogens are most commonly controlled with the chemical mancozeb or its derivatives. Metalaxyl, benomyl, iprobenfos, and copper (II) chloride and metalaxyl are other important fungicides used in the region. One of the most common bacterial diseases prevailing in the region is *Xanthomonas* wilt of banana caused by *X. campestris* pv. *musacearum*, but unfortunately there is no effective pesticide available to control this pathogen (**Table 1**). Viral plant pathogens responsible for cassava mosaic disease, cassava brown streak disease, banana bunchy top disease and maize lethal necrosis, also cause serious threat to food security in the region but here again, no efficient chemical control is available.

**Table 1:** Microbial pathogens affecting crop production in the GLCCA region and chemical pesticides used for their control.

Pathogens and pests	Species	Host plant	Impact^a^	Pesticides active ingredients	References
**Bacteria**	*Xanthomonas campestris* pv *musacearum*	Banana (*Musa* spp.)	***	NI	(Nyabyenda, 2006; Ndungo et al., 2008; Ndayihanzamaso et al., 2016; Nkuba et al., 2015; Rietveld et al., 2020)
	*Pseudomonas syringae* pv*. phaseolicola*, *P. syringae* pv*. syringae*	Bean (*Phaseolus vulgaris*)	***	Streptomycin sulphate	(Nyabyenda, 2005; Nkuba et al., 2015)
	*Xanthomonas campestris* pv*. phaseoli*		***	Streptomycin sulphate	(Nyabyenda, 2005)
	*Xanthomonas campestris* pv *manihotis*	Cassava (*Manihot esculenta*)	NI	NI	(Nyabyenda, 2005)
	*Ralstonia solanacearum*	Pepper (Chilli and sweet) (*Capsicum* sp.)	NI	NI	(Nyabyenda, 2006; Minengu et al., 2018)
	*Pseudomonas solanacearum*	Potato (*Solanum tuberosum*)	***	NI	(Nyabyenda, 2005)
	*Ralstonia solanacearum*		NI	NI	(Harahagazwe et al., 2007; Munyuli et al., 2017; Okonya et al., 2019a; Sharma et al., 2020)
	*Pseudomonas fuscovaginae*	Rice (*Oryza sativa*)	***	Formol	(Nyabyenda, 2005)
	*Clavibacter xyli*	Sugar cane (*Saccharum officinarum*)	***	Methoxy-ethyl, mercury chloride	(Nyabyenda, 2006)
	*Xanthomonas vasculorum*		***	Methoxy-ethyl, mercury chloride	(Nyabyenda, 2006)
	*Xanthomonas albilineans*		*	NI	(Nyabyenda, 2006)
	*Ralstonia solanacearum*	Tomato (*Lycopersicon esculentum*)	***	Formol	(Nyabyenda, 2006)
	*Pseudomonas solanacearum*		***	NI	(Nyabyenda, 2006)
	*Corynebacterium michiganense*		***		(Nyabyenda, 2006)
**Fungi**	*Fusarium oxysporum* var f. sp. *cubense*	*Banana* (*Musa* spp.)	***	NI	(Nyabyenda, 2006; Ndayihanzamaso et al., 2020)
	*Armillaria mellea, Cordona musae*		*	NI	(Nyabyenda, 2006)
	*Cladosporium musae*		*	NI	(Nyabyenda, 2006)
	*Gloesporium musarum*		*	NI	(Nyabyenda, 2006)
	*Helminthosporium tolurosum*		*	NI	(Nyabyenda, 2006)
	*Mycosphaerella musicola*		*	NI	(Nyabyenda, 2006)
	*Stachylidium theobrome*		*	NI	(Nyabyenda, 2006)
	*Ascochyta phaseolarum*	Bean (*Phaseolus vulgaris*)	***	Mancozeb, benomyl, benlate	(Nyabyenda, 2005)
	*Colletotrichum lindemuthianum*		***	Benomyl, mancozeb, thiophanate-methyl, benlate	(Nyabyenda, 2005)
	*Fusarium solani* f. sp. pv *phaseoli*		***	NI	(Nyabyenda, 2005)
	*Mycovellosiella phaseoli*		***	Benomyl, mancozeb, thiophanate-methyl, benlate	(Nyabyenda, 2005)
	*Pseudocercospora griseola*		***	Benomyl, thiophanate-methyl, mancozeb	(Busogoro et al., 1999; Nyabyenda, 2005; Kijana et al., 2017; Farrow and Muthoni-Andriatsitohaina, 2020)
	*Pythium* spp.		***	NI	(Nyabyenda, 2005)
	*Rhizoctonia solani*		***	NI	(Nyabyenda, 2005)
	*Sclerotinia sclerotiorum*		***	NI	(Nyabyenda, 2005)
	*Thielaviopsis basicola*		***	NI	(Nyabyenda, 2005)
	*Uromyces appendiculatus*		***	Mancozeb, benlate	(Nyabyenda, 2005; Farrow and Muthoni-Andriatsitohaina, 2020)
	*Alternaria* spp.	Cabbage (*Brassica* spp.)	***	NI	(Nyabyenda, 2006)
	*Botrytis cinerea*		***	NI	(Nyabyenda, 2006)
	*Peronospora* spp.		***	Mancozeb	(Nyabyenda, 2006)
	*Sclerotinia* spp.	Carrot (*Daucus carota*)	***	NI	(Nyabyenda, 2006)
	*Cercospora hemingsii*	Cassava (*Manihot esculenta*)	NI	NI	(Nyabyenda, 2005)
	*Glomerella manihotis*		NI	NI	(Nyabyenda, 2005)
	*Hemileia vastatrix*	Coffea (*Coffea* spp.)	***	Copper oxychloride 50%, dithianon, triadimefon	(Nyabyenda, 2006)
	*Ascochyta* sp.*, Phoma* sp.		NI	NI	(Nyabyenda, 2006; MINAGRI-Burundi, 2018)
	*Cercospora coffeicola*		NI	NI	(Nyabyenda, 2006; MINAGRI-Burundi, 2018)
	*Colletotrichum coffeanum*		NI	Copper oxychloride 50%, dithianon, captafol, chlorotalonil	(Nyabyenda, 2006; MINAGRI-Burundi, 2018)
	*Rhizoctonia solani*		NI	NI	(Nyabyenda, 2006; MINAGRI-Burundi, 2018);
	*Golovinomcyces* spp.	Eggplant (*Solanum melongena*)	***	NI	(Nyabyenda, 2006)
	*Leveillura* spp.,		***	NI	(Nyabyenda, 2006)
	*Peronospora* spp.		***	NI	(Nyabyenda, 2006)
	*Exserohilum turcicum, Helminthosporium maydis*	Maize (*Zea mays*)	**	Thiram, thioral (thiram + mancozeb), benomyl	(Nyabyenda, 2005)
	*Puccina polysora*		*	NI	(Nyabyenda, 2005)
	*Ustilago zeae, Sphacelotheca reilina*		*	NI	(Nyabyenda, 2005)
	*Puccinia* sp.	Morella (*Solanum aethiopicum*)	NI	NI	(Nyabyenda, 2005)
	*Alternaria* spp.	Onion (*Allium cepa*)	***	Maneb, mancozeb	(Nyabyenda, 2005)
	*Peronospora* spp.		***	Maneb, mancozeb	(Nyabyenda, 2006)
	*Puccinia* spp.		***	Maneb, mancozeb	(Nyabyenda, 2006)
	*Nothopassalora personata (Syn. Cercospora personata), Cercospora arachidicola*	Peanut (*Arachis hypogaea*)	***	NI	(Nyabyenda, 2005)
	*Macrophomina phaseolina*		**	NI	(Nyabyenda, 2005)
	*Puccinia arachidis*		**	NI	(Nyabyenda, 2005)
	*Sclerotium rolfsii*		**	NI	(Nyabyenda, 2005)
	*Colletotrichum nigrum, C. capsici*	Pepper (Chilli and sweet) (*Capsicum* sp.)	***	NI	(Nyabyenda, 2005)
	*Rhizoctonia solani*	Potato (*Solanum tuberosum*)	***	NI	(Nyabyenda, 2005; Harahagazwe et al., 2007)
	*Pyricularia oryzae*	Rice (*Oryza sativa*)	***	Benomyl, thiram, iprobenfos, isoprothiolane	(REMA, 2011; Nabahungu and Visser, 2013; Kanyange et al., 2019; Liboga et al., 2020)
	*Cercospora oryzae*		**	NI	(Nyabyenda, 2005)
	*Pythium bebaryanum*		**	NI	(Nyabyenda, 2005)
	*Rhizoctonia solani*		**	NI	(Nyabyenda, 2005)
	*Sclerotium rolfsii*		**	NI	(Nyabyenda, 2005)
	*Gerlachia oryzae*		NI	NI	(Nyabyenda, 2005)
	*Helminthosporium oryzae*		NI	NI	(Nyabyenda, 2005)
	*Sarocladium oryzae*		NI	NI	(Nyabyenda, 2005)
	*Peronosclerospora sorghii*	Sorghum (*Sorghum bicolor*)	***	NI	(Nyabyenda, 2005)
	*Colletotrichum graminicola*		NI	NI	(Nyabyenda, 2005)
	*Exserohilum turcicum*		NI	NI	(Nyabyenda, 2005)
	*Puccina purpurea*		NI	NI	(Nyabyenda, 2005)
	*Sphacelotheca cruenta, S. reiliana, S. sorghi*		NI	NI	(Nyabyenda, 2005)
	*Puccinia* sp.	Spinach (*Basella alba*)	NI	Mancozeb	(Nyabyenda, 2006)
	*Colletotrichum falactum*	Sugar cane (*Saccharum officinarum*)	***	NI	(Nyabyenda, 2006)
	*Fusarium moniliforme*		***	NI	(Nyabyenda, 2006)
	*Ustilago scitaminea*		***	NI	(Nyabyenda, 2006)
	*Puccinia melanocephala*		*	NI	(Nyabyenda, 2006)
	*Sclerospora sacchari*		*	NI	(Nyabyenda, 2006)
	*Alternaria solani*	Sweet potato (*Ipomoea batatas*)	***	NI	(Nyabyenda, 2006)
	*Armillaria mellea*	Tea (*Camellia sinensis*)	***	NI	(Nyabyenda, 2006)
	*Rosellinia arcuate*		***	NI	(Nyabyenda, 2006)
	*Colletotrichum camelliae*		*	NI	(Nyabyenda, 2006)
	*Corticium salmonicolor*		*	NI	(Nyabyenda, 2006)
	*Pestalotiopsis theae*		*	NI	(Nyabyenda, 2006)
	*Fusarium oxysporum* f. sp. pv *lycopersici*	Tomato (*Lycopersicon esculentum*)	***	NI	(Nyabyenda, 2006)
	*Cladosporium fulvum*		***	NI	(Nyabyenda, 2006)
	*Alternaria solani*		***	Mancozeb, metalaxyl	(Nyabyenda, 2006)
**Oomyceta**	*Phytophthora infestans*	Potato (*Solanum tuberosum*)	***	Mancozeb and metalaxyl	(Biruma et al., 2007; Harahagazwe et al., 2007; Bararyenya et al., 2018; Okonya et al., 2019a)
	*Phytophthora infestans*	Tomato (*Lycopersicon esculentum*)	***	Mancozeb and metalaxyl	(Nyabyenda, 2006)
	*Pythium* spp.		***	NI	(Nyabyenda, 2006)
**Virus (and vector)**	African cassava mosaic virus “ACMV”, East African cassava mosaic virus “EACMV”, East African cassava mosaic virus-Uganda “EACMV-UG” (Vector: *Bemisia tabaci*)	Cassava (*Manihot esculenta*)	***	NI	(Bigirimana et al., 2004; Nyabyenda, 2005; Thresh and Cooter, 2005; Bigirimana et al., 2011)
	Cassava brown streak virus “CBSV”, Ugandan cassava brown streak virus “UCBSV”, Cassava root necrosis disease “CRND” (Vector: *Bemisia tabaci*)		***	NI	(Nyabyenda, 2005; Bigirimana et al., 2011; Maruthi et al., 2017; Munganyinka et al., 2018; Okonya et al., 2019a; Muhindo et al., 2020);
	Banana bunchy top virus “BBTV” (Vector: *Pentalonia nigronervosa*)	Banana (*Musa* spp.)	***	NI	(Gaidashova et al., 2010; Niyongere et al., 2013; Boloy et al., 2014; Mukwa et al., 2014; Okonya et al., 2019a; Raut and Ranade, 2004)
	Sweet potato chlorotic stunt virus “SPCSV” *(Vector: Bemisia tabaci)*, Sweet potato feathery mottle virus “SPFMV” (Vector: *Myzus persicae* and *Aphis gossypii*)	Sweet potato (*Ipomoea batatas*)	***	NI	(Sheffield, 1957; Nyabyenda, 2005)
	Bean common mosaic virus “BCMV” (Vector: Aphid)	Bean (*Phaseolus vulgaris*)	***	Dimethoate	(Nyabyenda, 2005);
	Virus A, Virus X, Virus S, Virus Y *(Vector: Aphid)*	Potato (*Solanum tuberosum*)	***	NI	(Nyabyenda, 2005)
	Groundnut rosette virus “GRV” (Vector: *Aphis craccivora*)	Peanut (*Arachis hypogaea*)	***	NI	(Nyabyenda, 2005)
	Maize chlorotic mottle virus “MCMV” (Vectors: Thrips, root worms and leaf beetles), Maize streak virus “MSV” (Vector: *Cicadulina* spp.) Maize lethal necrosis “MLN” (Maize chlorotic mottle virus and Sugarcane mosaic virus)	Maize (*Zea mays*)	***	NI	(Nyabyenda, 2005; Adams et al., 2014; Lukanda et al., 2014; Mahuku et al., 2015; Isabirye and Rwomushana, 2016; Redinbaugh and Stewart, 2018; Casinga et al., 2021)
	Pepper mild mottle virus (Vector: Thrips)	Pepper (Chilli and sweet) (*Capsicum* spp.)	***	NI	(Nyabyenda, 2006)

*, **, ***: slight, moderate and high impact, respectively; NI, No Information.

Insects, nematodes, and weeds are also responsible for severe yield losses. Important insect pests affecting major crops in the region include fall armyworm (*Spodoptera frugiperda*), tomato leafminer (*Tuta absoluta*), coffee bugs (*Antestiopsis orbitalis ghesquierei*), whitefly (*Bemisia tabaci*), banana aphid (*Pentalonia nigronervosa*), and coton aphid (*Aphis gossypii*) (Nyabyenda, 2005; Dushimirimana et al., 2016; MINAGRI-Burundi, 2018; Niyibizi et al., 2019; Belga, 2020; Mukwa et al., 2020; Cokola et al., 2021; Niassy et al., 2021). The control of these insects relies heavily on the use of synthetic products such as acephate against *S. frugiperda* and *T. absoluta*, chlorpyrifos-ethyl against *A. orbitalis ghesquierei* and imidacloprid against aphids. Other major insecticides used in the region include lambda-cyhalothrin, cypermethrin, dimethoate, pirimiphos-methyl + permethrin, metaldehyde, abamectin and emamectin benzoate (MINAGRI-Burundi, 2018; ARECO-Rwanda Nziza, 2020; GUCE, 2022). Several species of nematodes also damage plants, including *Meloidogyne javanica* on various crops, *Pratylenchus goodeyi* and *Helicotylenchus multicinctus* on banana plants, and *Ditylenchus* spp. on potatoes (Coyne et al., 2018). These nematode-caused diseases are managed by using chemicals such as dazomet and terbufos. Parasitic plants or weeds that compete with crops such as sugarcane, rice, sorghum, and maize are also problematic and include among others *Striga* spp., *Cyperus* spp. and *Echinochloa* spp. Their control involves chemical herbicides like glyphosate, atrazine and dalapon (Nyabyenda, 2005; Rodenburg et al., 2016; Runo and Kuria, 2018).

The spread of some of these diseases and pests has led to historic famines in the GLCCA. For example, the Integrated Food Security Phase Classification (IPC) reported that nearly 13.1 million people in the DRC were acutely food insecure due to the spread of the fall armyworm on maize crops (MINAGRI-RDC, 2018; IPC, 2021). In 2004-2005, the outbreak of cassava mosaic disease led to severe food shortages that threatened many families in the north-eastern provinces of Kirundo and Muyinga in Burundi. As a result, 100 famine-related deaths were reported (Legg et al., 2006).

## 3 Limitations in local regulation of phytosanitary products and associated risks

Pesticide on the market must be regularly reconsidered by the relevant registration authorities and subsequently included in a list of approved or banned products. The aim is to ensure the quality, efficacy and safety of the chemicals used. However, there are some specific features of the management of pesticides in the GLCCA region that deserve special attention. First, many chemical pesticides banned in the European Union (EU Food Safety, 2023) are still officially registered in the GLCCA countries, representing up to 30% (**Table 2**) of the total number of products legally distributed (**Table S1**). It includes the highly hazardous but most commonly used fungicide mancozeb and the insecticides imidacloprid, acephate and dimethoate banned in EU (Lewis et al., 2016). Furthermore, some ingredients such as endosulfan and dichlorodiphenyltrichloroethane, which have been officially discarded by governments in the GLCCA region, are still available in local markets (Ngweme et al., 2019; Bassily, 2022). Second, malpractices in the handling of chemical pesticides are common, either among traders or end-users such as farmers or industrial workers. These malpractices include the sale of adulterated products, failure to use personal protective equipment, incorrect dosage and selection of products, and application at inappropriate times (Wipfler and ter Host, 2018; Okonya et al., 2019b; Balasha et al., 2023). One of the consequences is that residues of pesticides have been found at toxic levels in crops and vegetables such as tomato and amaranth (Kavatsurwa et al., 2014; Ngweme et al., 2021; Ndisanze et al., 2022).

**Table 2:** Status of some EU banned chemical pesticides in GLCCA.

Class of pesticides	Active ingredients banned in EU	Chemical family	Regulation status		
			Burundi	DRC	Rwanda
**Insecticides**	Acephate	Organophosphorus	R	NI	NI
	Benfuracarb	Carbamates	R	NI	NI
	Carbosulfan	Carbamates	R	NI	NI
	Chlorfenapyr	Pyrroles	NI	NI	R
	Chlorpyrifos	Organophosphorus	R	R	R
	Cyfluthrin	Pyrethroids	R	NI	NI
	Diafenthiuron	Thioureas	NI	NI	R
	Diazinon	Organophosphorus	R	B	B
	Dichlorvos	Organophosphorus	R	BU	B
	Dimethoate	Organophosphorus	R	R	B
	Endosulfan	Organochlorines	B	BU	B
	Fenbutatin oxide	Organometallics	NI	NI	R
	Fenitrothion	Organophosphorus	R	R	NI
	Fenthion	Organophosphorus	R	NI	B
	Fenvalerate	Pyrethroids	R	NI	NI
	Fipronil	Phenylpyrazole	NI	R	R
	Flufenoxuron	Benzoylureas	NI	NI	R
	Imidacloprid	Neonicotinoid	R	R	R
	Isoxathion	Organophosphorus	R	NI	NI
	Omethoate	Organophosphorus	R	NI	NI
	Oxydemeton-methyl	Organophosphorus	R	NI	NI
	Permethrin	Pyrethroids	R	NI	R
	Profenofos	Organophosphorus	R	NI	R
	Pirimiphos-methyl	Organophosphorus	R	NI	NI
	Pymetrozine	Triazine	NI	NI	R
	Tetradifon	Diphenyl	NI	NI	R
	Tetramethrin	Pyrethroids	NI	R	NI
	Thiacloprid	Neonicotinoid	NI	NI	R
	Tralomethrin	Pyrethroids	R	NI	NI
	Triazophos	Organophosphorus	R	NI	NI
**Herbicides**	Atrazine	Triazine	R	NI	B
	Dalapon	Organochlorines	R	NI	R
	Hexazinone	Triazine	R	NI	NI
	Linuron	Methylureas	NI	NI	R
	Oxadiazon	Oxadiazole	NI	R	NI
	Paraquat	Pyridine	R	BU	B
	Propanil	Anilide	NI	NI	R
	Terbutryn	Triazine	NI	NI	R
**Fungicides**	Benomyl	Carbamates	R	NI	R
	Bitertanol	Triazole	NI	NI	R
	Carbendazim	Carbamates	NI	BU	R
	Chlorothalonil	Organochlorines	R	BU	R
	Cyproconazole	Triazole	NI	NI	R
	Epoxiconazole	Triazole	NI	NI	R
	Fenamidone	Imidazolinone	NI	NI	R
	Fenarimol	Pyrimidine	NI	NI	R
	Iprobenfos	Organophosphorus	R	NI	NI
	Iprodione	Dicarboximides	R	NI	R
	Mancozeb	Thiocarbamates	R	R	R
	Pencycuron	Phenylureas	NI	NI	R
	Propineb	Carbamates	NI	NI	R
	Triadimenol	Triazole	NI	R	NI
	Thiram	Thiocarbamate	R	R	R
**Rodenticides**	Bromadiolone	Coumarin	NI	R	NI
	Brodifacoum	Coumarin	R	BU	R
	Chlorophacinone	Indandione	R	NI	NI
	Coumatetralyl	Coumarin	R	NI	R
	Diphacinone	Indandione	R	NI	R
**Insecticide-nematicides**	Carbofuran	Carbamates	R	NI	R

R, Registered; B, Banned; NI, No Information; NRU, Not Registered but in Use; BU, Banned but in Use.

Consequently, some acute symptoms and even death of humans and animals due to pesticide poisoning have been reported in the region. These symptoms include reddened eyes, itchy skin, teary eyes, burning eyes, runny nose, headache, difficulty breathing, and heavy sweating (Ndayambaje et al., 2019; Okonya et al., 2019b; Balasha et al., 2023). Chronic effects of pesticide exposure, including cancer, infertility, and neurological problems (Mahmood et al., 2016), may be a reality in the region or may occur in the near future, although no official survey has been conducted to date. Several reasons may explain this chaotic situation in the region such as poverty, ignorance, and illiteracy among users (Niyongere et al., 2015; Muliele et al., 2018; Ndayambaje et al., 2019; Balasha et al., 2023), inadequate governance in the pesticide sector, and possible socio-economic influence of agrochemical companies on officials (Gaberell and Viret, 2022).

## 4 Non-biological alternatives to chemical pesticides in GLLCA

The adverse effects of chemical pesticides have prompted the worldwide scientific community and policy makers to search for alternatives and promote the so-called IPM, which is defined as a holistic approach that aims to control plant pests and diseases by mobilizing all existing methods while reducing reliance on chemicals (Stenberg, 2017). IPM promotes the development of non-biological strategies such as prevention, monitoring, and rational use of chemical phytoprotectants products, and the implementation of biological control methods, which will be discussed later.

Good agricultural practices are preventive measures consisting, for example, in the use of certified varieties, crop rotation, field sanitation and balanced fertilization. These practices have been adopted to some extent in the GLCCA region and promising results have been obtained. For example, cassava mosaic disease and maize streak disease have been controlled using resistant varieties (Legg et al., 2006; Nkurunziza et al., 2012). The banana bunchy top disease has recently been reduced via macro- and micropropagation of healthy suckers (Niyongere et al., 2013; Tchatchambe et al., 2019; Paka et al., 2021). The incidence of banana *Xanthomonas* wilt and potato bacterial wilt caused by *R. solani* has been limited via crop rotation, field sanitation, sterilization of farm tools, and avoidance of their exchange (Harahagazwe et al., 2007; Ndayihanzamaso et al., 2016; Uwamahoro et al., 2020). However, despite many attempts involving certified varieties or other agricultural practices, no substantial results have been achieved to control fungal diseases in the region using these approaches. Moreover, implementation of good agricultural practices is unfortunately hampered by several factors, including demographic pressure on arable land, the relative time required to develop new varieties, the lack of durability of resistance in developed varieties, and the consequences of climate change in the agro-ecological context (Pandit et al., 2022).

Phytosanitary products must be used in a rational way, taking into account the epidemiological factors of the specific pathogens, which are usually obtained from accurate field monitoring. Where chemicals are irreplaceable, careful consideration must be given to the dose applied and the less toxic products must be favored. For example, flupyradifurone has been proposed to replace the insecticide imidacloprid (Maloney et al., 2020) and copper hydroxide in substitution of mancozeb (FPS Health, 2023) in EU. Unfortunately, this practice is less widespread in the region, where the choice of pesticide is made at random without prior information on the disease status and on recommended products for optimal efficiency.

## 5 Implementation of biological control

Biological control is a promising approach for plant protection based on the use of living organisms and/or their derivatives. These include macroorganisms (predatory insects, mites, and nematodes), microorganisms (beneficial bacteria, fungi, protozoa, viruses, yeasts) and biochemicals (semiochemicals, plant extracts, plant growth regulators and organic acids) (DunhamTrimmer, 2023). The biocontrol market accounts for 10% of the global pesticide market, with North America leading the way in promoting BCPs, while African share is very insignificant led by countries like Nigeria, South Africa, and Kenya (Marrone, 2023; Mordor Intelligence, 2023). Biological control is a new paradigm in GLCCA and only seven biocontrol products (3%) are registered up to now (**Table S1**).

### 5.1 Macroorganisms

Macroorganisms are either predators or parasitoids of the same kind of pathogenic organisms (insects, mites, or nematodes). They are used in the form of eggs, larvae, pupae, or adults to kill or parasitize the target pest (Ramalakshmi et al., 2020; DunhamTrimmer, 2023). Insect predators have been successfully used in the region to control some pests. For instance, *Epidinocarpis lopezi* isolated in South America, has been used to control the cassava mealy bug (*Phenacoccus Manihoti*) (Neuenschwander, 2001; Nyabyenda, 2005), while *Gyranusoidea tebygi* and *Anagyrus mangicola* have been developed to control the devastating mango mealy bug in Burundi (FAO, 2022). Other common predators could be introduced locally to control a range of pests. Indeed, the green lacewing (*Chrysoperla carnea*), the Malaysian ladybird beetle (*Chilocorus nigritus*) and the mealybug ladybird (*Cryptolaemus montrouzieri*) have been reported to be effective in controlling the cotton aphid jassid, peanut thrips, sugarcane scales and coffee and mango mealybugs respectively (Ramalakshmi et al., 2020).

### 5.2 Biochemicals

Biochemicals in the form of plant extracts appear to be the most widely used biocontrol agents in the region. These extracts are prepared from various plant species like *Azadirachta indica*, *Capsicum* spp., *Allium sativum*, *Tephrosia* spp., *Tithonia diversifolia* or *Ricinus communis* and are used as insecticides to control various pests such as *S. frugiperda* on maize, *Tuta absoluta* on tomato, *Ophiomya phaseoli*, *Aphis fabae* on beans, etc. (Rutikanga, 2015; PES, 2021; Korangi Alleluya et al., 2021). In addition, two plant molecules azadirachtin and pyrethrin, and spinosad from actinomycetes have been registered as insecticides in Rwanda (**Table S1**). However, the use of these extracts is still globally rudimentary and based on traditional and community-based knowledge. Moreover, the use of this type of products requires increased land for growing plants to prepare the extracts, which would inevitably compete with staple and cash crops for agricultural land.

### 5.3 Microorganisms

Several microorganisms encompassing bacteria, fungi, and yeasts are commercialized as biocontrol agents. Fungal based agents mainly include the genera *Trichoderma*, *Metarhizium* and *Beauveria*; while yeasts include the genera *Pichia*, *Yarrowia* and *Saccharomyces*. The largest group of worldwide marketed microorganisms are bacteria (up to 75%), dominated by plant growth-promoting rhizobacteria (PGPR) of the genera *Bacillus*, *Pseudomonas* and *Streptomyces* (Saeed et al., 2021; Bonaterra et al., 2022; Dimkić et al., 2022). However, these globally adopted microbial-based alternatives are rarely used in the GLCCA region, and mainly as bioinsecticides such as *B. thuringiensis* sold in the three countries and *B. bassiana* and *T. harzanium* only registered in Rwanda. Here, an overview of the PGPR reported in literature with biocontrol activities and their mechanisms of action is presented for their possible integration into local agricultural systems in GLCCA.

## 6 Plant growth-promoting rhizobacteria in crop protection

Plant growth-promoting rhizobacteria are a group of bacteria found mainly in the vicinity of plant roots, the rhizosphere. The plant provides them with nutrients in the form of root exudates, while these bacteria protect the host and promote its growth. These benefits are mediated through various pathways, including biofertilization by nitrogen fixation and phosphate solubilization, phytostimulation by the production of phytohormones (cytokinin, abscisic acid, auxin, gibberellins, and ethylene), stress tolerance and biological control (Vejan et al., 2016; Anckaert et al., 2021; El-Saadony et al., 2022). PGPR include several species belonging to different genera, namely *Rhizobium*, *Azotobacter*, *Streptomyces*, *Enterobacter*, *Klebsiella*, *Rhodococcus*, *Paenibacillus*, *Variovorax*, *Azosprillum*, *Bulkholderia*, *Serratia*, *Pseudomonas* and *Bacillus* (Caulier et al., 2018). Bacterial strains of the genera *Streptomyces*, *Paenibacillus*, *Pseudomonas* and *Bacillus* are the best described and have been shown to be effective biocontrol agents against various plant pathogens through their multiple mechanisms of action such as nutrient and niche competition, antibiosis, signal interference and induction of host resistance (Wang et al., 2021).

### 6.1 Competition

Competition for nutrients and niches between pathogens and beneficial microbes is an important factor in limiting disease incidence and severity (Bishnoi, 2015). Indeed, rhizosphere microorganisms compete for a variety of plant root exudates, including sugars, amino acids and organic acids. These exudates also act as chemoattractants for bacteria to plant roots. These beneficial bacteria colonize the root and can inhibit the growth of soil-borne phytopathogenic microbes by competing for nutrients such as glucose, asparagine, and iron (Dutta and Lee, 2022). Besides exudates, competition for iron is the most well-studied mechanism and involves several bacterial siderophores, such as hydroxamates (*Pseudomonas* spp.), carboxylates (*Rhizobium* spp.) and catecholates (*Pseudomonas* spp., *Bacillus* spp.), which are used to extract iron from insoluble forms and sequester it from other competitors by chelation (Arguelles-Arias et al., 2009; Mazumdar et al., 2020). On the other hand, rhizobacteria outcompete other microbes for space through their ability to form biofilms, which also act as shelter-like against pathogens (Flemming et al., 2016; Singh et al., 2022).

### 6.2 Antibiosis

Antibiosis is a biological process in which antimicrobial substances synthesized by microorganisms inhibit or kill plant pathogens (Fira et al., 2018). These are toxins that interfere with the synthesis of the cell membrane or other metabolic processes of the pathogen and are variable depending on the PGPR species. For example, *Pseudomonas* spp. are known to produce 2,4-diacetylphloroglucinol, phenazine, pyoluteorin, pyrrolnitrin, hydrogen cyanide and a wide range of lipopeptides such as viscosin, amphisin, orfamide, bananamide and sessilin (Wang et al., 2021; Oni et al., 2022). *Bacillus* spp. produce various cyclic lipopeptides (CLPs), polyketides, dipeptides, RiPPs and volatile organic compounds (Arguelles Arias et al., 2013; Caulier et al., 2019; Anckaert et al., 2021). *Streptomyces* species are known producers of several antibiotics such as kasugamycin (antifungal and antibacterial), polyoxin B and D, validamycin (antifungal) families (Barka et al., 2016), as well as cyclic lipopeptides such as lipopeptins recently discovered with antagonistic activity against *Fusarium oxysporum* (Wang et al., 2023). *Paenibacillus* spp. are another important PGPR, producing mainly polymyxin active against Gram-negative bacteria, fusaricidin with anti-Gram-positive bacteria and antifungal activities (Ali et al., 2021), pelgipeptins and tridecaptins with antagonistic activity against fungi and bacteria (Le et al., 2020; Costa et al., 2022). PGPR also produce lytic enzymes (the so-called cell wall degrading enzymes) such as chitinase, glucanase, cellulase, xylanase, and pectinase, which are known for their antagonistic activities against pathogens (Abdelaziz et al., 2023).

### 6.3 Signal interference

Like other organisms, bacteria live in communities where they communicate to obtain nutrients and perform other metabolic functions. Cell-to-cell communication within bacteria is known as quorum sensing (QS), mediated by several molecules including N-acylhomoserine lactones, oligopeptides and LuxS/autoinducers (De Kievit, 2009; Rodríguez et al., 2020; Wang et al., 2021). QS is important for bacterial phenotype and virulence, biofilm formation and other physiological processes. QS-mediated metabolites from phytopathogenic bacteria can be chemically or enzymatically degraded by so-called quorum quenching (QQ) enzymes such as lactonase, acylase and oxidoreductase produced by some PGPR. This interferes with QS-regulated processes that are crucial for pathogen development (Sikdar and Elias, 2020) and some important bacterial diseases such as *Pectobacterium carotovorum*, *Pseudomonas syringae*, *Ralstonia solanacearum*, *Erwinia amylovora* have been successfully managed by using this approach (Rodríguez et al., 2020).

### 6.4 Induced systemic resistance

Induced systemic resistance (ISR) is the process by which plants treated with PGPR species or their secreted molecules induce defense genes and develop immunity to subsequent plausible pathogen attack. PGPR-induced ISR is phenotypically similar to the well-studied systemic acquired resistance (SAR), which is activated after a first infection by an incompatible or necrotizing pathogen (Pršić and Ongena, 2020). This process is regulated by the jasmonate or ethylene phytohormone pathways, whereas SAR is dependent on the salicylic acid regulation pathway. The main elicitors of ISR produced by PGPR are Acyl-Homoserine Lactones, cyclic lipopeptides, rhamnolipids, N-alkylated benzylamine derivative, siderophores, volatile compounds and other metabolites with antibiotic functions such as 2,4-diacetylphloroglucinol and phenazine (Pršić and Ongena, 2020; Wang et al., 2021; Zhu et al., 2022). These elicitors act as microbial associated molecular patterns and are perceived by specific transmembrane pattern recognition receptors, as it has been shown for the pathogen triggered-SAR. However, for a successful symbiosis with plant roots, PGPR have evolved to evade or suppress the pattern triggered immunity developed by the plant as the first line of defense (Yu et al., 2022; Seth et al., 2023). This PGPR-induced plant immunity ultimately leads to defense mechanisms such as cell wall reinforcement via deposition of lignin or callose, enhanced production of defense-related proteins (lipoxygenase, glucanase, chitinase, phenylalanine ammonia-lyase) and accumulation of phytoalexins as small-size antimicrobial metabolites (Abdelaziz et al., 2023).

## 7 *Bacillus* as successful PGPR for biological control

*Bacillus* is a rod-shaped, gram-positive and spore-forming bacterium that is widespread in a variety of ecological niches (Yin et al., 2023). Phylogenetically, the *Bacillus* genus can be broadly divided into two clades, namely the *Bacillus cereus* group, which includes the well-known and most commercialized bioinsecticide *B. thuringiensis*, and the *Bacillus subtilis* group, which includes most of *Bacillus* strains marketed for the biocontrol of microbial pathogens (Dunlap, 2019; Nikolaidis et al., 2022). Strains in the *B. subtilis* group are generally recognized as safe and have therefore been used as alternatives to chemical pesticides in agriculture (Etesami et al., 2023). For example, *B. licheniformis* SB3086 (Ecoguard^®^, Novozymes^®^, Biofungicide^®^, Green Relief^®^), *B. pumilus* GB34 (GB34^®^, Concentrated Biological Fungicide^®^, Ballad^®^), *B. velezensis* MBI600 (Subtilex^®^, Histick N/T^®^), *B. velezensis* GBO3 (Kodiak^®^, Companion^®^), *B. velezensis* QST713 (Serenade^®^, Rhapsody^®^) and *B. velezensis* FZB42 (Taegro^®^, Rhizovital^®^) are among the commercial biocontrol agents from the *B. subtilis* group (Lahlali et al., 2022; Yadav et al., 2022). These strains are continuously attracting researchers and industrials thanks to their intrinsic ability to form resistant endospores that allow stable formulations, relatively rapid growth on different substrates, and the secretion of a wealth of bioactive secondary metabolites retaining key biocontrol functions (Prasad et al., 2023). The biosynthesis of these biochemicals involves up to 10% of the bacterial genome and includes a huge diversity of compounds produced via non-ribosomal and ribosomal pathways (Grubbs et al., 2017; Borriss, 2020; Yin et al., 2023).

Cyclic lipopeptides are a group of compounds with a fatty acid and peptide moiety that are produced via the non-ribosomal pathway involving a multi-modular enzyme complex (Iqbal et al., 2023). This machinery gives rise to a large chemical diversity with different structures, based on their amino acid sequence or on the length of the fatty acid chain. These metabolites are classified into the heptapeptides surfactins (pumilacidins/lichenisins) and iturins (iturin A, mycosubtilin, bacillomycin) and the decapeptides fengycins (plipastatins); together with their homologues of different alkyl chain lengths (C_12_ - C_17_ for surfactins, C_13_-C_17_ for iturins and C_14_-C_18_ for fengycins) (Théatre et al., 2022). They are produced by members of the *Bacillus subtilis* group and are known to be involved in the induction of resistance in plants (Pršić and Ongena, 2020; Dimkić et al., 2022; Mahapatra et al., 2022) and, thanks to their amphiphilic nature, exhibit antimicrobial activities against a wide range of pathogenic microorganisms by penetrating and disrupting the cell membrane of target organisms (Fira et al., 2018; Ngalimat et al., 2021; Puan et al., 2023).

Polyketides are bioactive metabolites synthetized via pathways catalyzed either by the polyketide synthases (difficidins and macrolactins) or by the hybrid polyketide synthases/non-ribosomal peptide synthetases (bacillaene) and are structurally composed of an alternation of polyenes and carbonyl groups (Iqbal et al., 2023). They retain consistent antibacterial activity against several phytopathogens either by interfering with protein synthesis or by damaging the bacterial cell wall (Fazle Rabbee and Baek, 2020; Miao et al., 2023; Zhang N. et al., 2023). The siderophore bacillibactin is a catecholate peptide also produced via the non-ribosomal pathway that retains a high affinity for iron. This property is very important in bacterial niche competition, helping bacilli to gain advantage over their competitors by chelating available iron in the environment (Yu et al., 2011). The dipeptide bacilysin is biosynthesized via a typical non-ribosomal pathway not shared by the lipopeptide/polyketide/siderophore trio. It exhibits toxicity against bacteria by blocking peptidoglycan biosynthesis (Borriss, 2015) and against fungi by probably inhibiting fungal glycans in a manner similar to the inhibition of bacterial peptidoglycan synthesis (Han X. et al., 2021).

Ribosomally produced and post-translationally modified peptides include bacteriocins and lantibiotics (such as amylocyclicin, amylolysin, plantazolicin, subtilin, pumilarin or LCI) known for their high inhibitory potential against bacterial pathogens, either by vascularization of the protoplasm or by pore formation or cell disruption (Sumi et al., 2015). In addition to these soluble metabolites, *Bacillus* volatile compounds (hydrocarbons nonane and tridecane, acetoin, 2,3-butanediol, benzaldehyde, 3-methylpropanoic acid, methylbenzene, benzothiazole, etc.) have been reported to inhibit the growth of some fungi, bacteria, and nematodes by disrupting cell wall integrity (Caulier et al., 2019; Kai, 2020; Iqbal et al., 2023).

## 8 The potential of *Bacillus*-based biocontrol in the GLCCA region, a myth or reality

The market for biocontrol agents based on strains of the *B. subtilis* group for the control of plant diseases is growing rapidly around the world but no product based on this bacterium is registered in the region. However, some promising preliminary studies using *B. velezensis* strains to control fungal diseases in Burundi and DRC have demonstrated the potential of these bacteria to adapt to local agro-ecological conditions, which could pave the way for their possible future dissemination and adoption by local farmers. *Bacillus velezensis* S499, isolated in Ituri/DRC in 1950s (Delcambe and Devignat, 1957), is effective against *Fusarium* sp. on tomato (65-70% of disease reduction) under field conditions in Burundi, and lipopeptide-mediated ISR is proposed as the main involved mechanism (Nihorimbere et al., 2010). Lipopeptides secreted by the same strain showed high *in vitro* antagonistic activity against pathogens isolated from maize cob such as *Rhizopus stolonifer, Penicillium variable, Fusarium verticillioides*. In the same study, S499 was able to efficiently reduce disease severity (39-67%) caused by these fungi in greenhouse and field conditions (Kulimushi et al., 2018). In addition, *B. velezensis* GA1 and its cyclic lipopeptides played an important role in the control of peanut stem rot disease caused by *Athelia rolfsii*, providing up to 60% of protection (Korangi Alleluya et al., 2023).

Furthermore, a number of studies have demonstrated the potential of *Bacillus* isolates in controlling a range of important and widespread phytopathogens reported in the GLCCA. For example, *B. altitudinis* and *B. velezensis* were shown to control rice blast caused by *Pyricularia oryzae* (15% and 25% of disease reduction, respectively) by direct antagonism and ISR involving iturin and fengycin (Lam et al., 2021). Volatiles from *B. amyloliquefaciens* NJN-6 reduce the mycelial growth of the banana pathogen *F. oxysporum* f.sp. *cubense in vitro* by up to 30-40% (Yuan et al., 2012). *B. subtilis* AUBB20 inhibited the growth of the coffee pathogen *F. xylarioides* through its secreted lytic enzymes (Muleta et al., 2007). Antagonistic activities of *Bacillus subtilis* strains have also been reported against fungi and oomycetes, including *Alternaria linariae* (da Silva Junior et al., 2023), *Sclerotinia sclerotiorum*, *Rhizoctonia solani* (Al-Mutar et al., 2023), *Colletotrichum lindemuthianum* (Martins et al., 2019), *Mycosphaerella fijiensis* (Gutierrez-Monsalve et al., 2015), *Verticillium dahliae* (Dhouib et al., 2019), *Fusarium oxysporum* f. sp. *lycopersici* (Aydi Ben Abdallah et al., 2017), *Colletotrichum gloeosporioides* (Luna-Bulbarela et al., 2018) and *Phytophthora infestans* (Zhang J. et al., 2023b).

The biocontrol activity of *Bacillus* has also been demonstrated against important bacterial phytopathogens. For example, the application of *B. subtilis* to potato plants infected with *R. solanacearum* reduced the incidence of wilt by about 50% (Elazouni et al., 2019). Tomato bacterial wilt caused by the same bacterium was efficiently controlled by *B. methylotrophicus* DR-08 due to its secreted metabolites oxydifficidin and difficidin (Im et al., 2019). *Bacillus megaterium* USB2103 efficiently controlled common bean bacterial wilt caused by *Xanthomonas axonopodis* pv. *phaseoli* through ISR mechanisms (Giorgio et al., 2016). Brown sheath blight of rice caused by *Pseudomonas fuscovaginae* was successfully controlled (76.6%) by *B. amyloliquefaciens* Bk7 (Ullah Kakar et al., 2014). Other examples of *Bacillus* spp. with potential applications in the control of fungal and bacterial diseases for crop protection in the GLCCA are shown in **Table 3**.

**Table 3:** Some global success stories of *Bacillus* isolates (*subtilis* group) in biocontrol of important crop pathogens reported in the GLCCA.

*Bacillus* strains	Plant	Pathogen	Mechanism involved	Experiment condition	Biocontrol potential	References
***B. velezensis* NJN-6**	Banana	*Fusarium oxysporum* f.sp. *cubense*	Antibiosis (VOCs)	*In vitro*	30-40% of *Foc* inhibition compared to control	(Yuan et al., 2012)
***B. velezensis* EB1**			Antibiosis and ISR	*In vitro, in greenhouse*	75.43% Inhibition rate	(Xiang et al., 2023)
***B. amyloliquefaciens* W19**			Antibiosis(CLPs and VOCs)	*In vitro, in greenhouse*	21% Inihibition rate	(Wang et al., 2013)
***B. siamensis* Gxun-6**			Antibiosis (probably), ISR(probably)	*In vitro, in greenhouse*	>68.8% Inhibition rate; >88.26% Biocontrol efficacy	(Shen et al., 2022)
***B. pumilus* CCIBP-C5**		*Mycosphaerella fijiensis*	Antibiosis	*In vitro, in greenhouse*	45% Inhibition rate; 33.6% Biocontrol efficacy	(Di Francesco et al., 2017)
***B. licheniformis, B. siamensis, B. subtilis* subsp. *Inaquosorum***			ISR	*In greenhouse*	ns	(Marcano et al., 2016)
***B. velezensis* QST713**	Bean	*Xanthomonas axonopodis* pv. *phaseoli*	Antibiosis (PKs)	*In vivo*	52.26% Disease incidence reduction	(Belete et al., 2021)
***B. pumilus***		*Sclerotium rolfsii*	ns	*In greenhouse*	26% Disease incidence reduction	(Pleban et al., 1995)
***B. velezensis* MBI600, *B. velezensis* FZB42*, B. velezensis* QST713**		*Colletotrichum lindemuthianum*	ISR (probably)	*In greenhouse*	21.9%,15.2%, 10.9% Diseased plants respectively	(Tinivella et al., 2009)
***B. velezensis* GBO3**	Cassava	*Fusarium solani*	Antibiosis	*In vitro, in greenhouse*	Approx. 10mm inhibition zone; 0% Disease incidence (asymptomatic plants)	(Freitas et al., 2019)
***B. subtilis* ME9**		*Xanthomonas phaseoli pv. manihotis*	Antibiosis	*In vitro, in greenhouse*	Approx. 14mm inhibition zone diameter	(Feng et al., 2023)
***B. subtilis* AUBB20**	Coffea	*Fusarium xylarioides*	Antibiosis (lytic enzymes)	*In vitro*	Approx. 30-70 mm inhibition zone	(Muleta et al., 2007)
***B. pumilus***	Cotton	*Rhizoctonia solani*	ns	*In greenhouse*	56% Disease incidence reduction	(Pleban et al., 1995)
***B. velezensis* SQR9**	Cucumber	*Fusarium oxysporum* f.sp. *cucumerinum*	Competition	*In vitro, in greenhouse*	49-61% Disease reduction compared to control	(Cao et al., 2011)
***B. subtilis Y*B-04**			Antibiosis (lytic enzymes), Competition for nutrients (siderophore)	*In vitro, in greenhouse*	>90% Control efficacy	(Xu et al., 2022)
***B. subtilis* TM4**	Maize	*Fusarium verticolloïdes*	ns	*In field*	<9% Disease incidence	(Mirsam et al., 2021)
***B. licheniformis***	Mango	*Colletotrichum gloeosporioides Botryosphaeria* spp.	Antibiosis	*In vivo* (on harvested fruits)	30-40% Disease incidence	(Govender et al., 2005)
***B. safensis* C3**	Mungo bean	*Xanthomonas axonopodis* *Pseudomonas syringae*	Antibiosis (Bacteriocin-like antimicrobial peptides)	*In vitro*	ns	(Romero-Severson et al., 2021)
***B. subtilis* BsW4, Bs76, *B. amyloliquefaciens* Ba100**	Pea	*Ascochyta pinodes*	Antibiosis (CLPs)	*In vitro, in greenhouse* and *in field*	>65% Biocontrol efficacy	(Liu et al., 2016)
***B. velezensis* TN-TB4, *B. amyloliquefaciens* TN-TB6**	Peanut	*Cercospora arachidicola*	Antibiosis	*In vitro*	>55% Inhibition rate	(Thanh & Yen, 2023)
***B. subtilis* G1**		*Macrophomina phaseolina*	Antibiosis and ISR (hypothesis)	*In greenhouse, in field*	15-20% Disease incidence	(Shifa et al., 2018)
***B. velezensis* LHSB1**		*Sclerotium rolfsii*	Antibiosis (CLPs)	*In vitro, in greenhouse*	93.8% Inhibition rate; 62.6–70.8% Biocontrol efficacy	(Chen L. et al., 2019)
***B. velezensis* 6-5**	Potato	*Phytophtora infestans*	Antibiosis (lytic enzymes)	*In vitro*	>90% Inhibition rate	(Zhang J. et al., 2023a)
***B. amyloliquefaciens* Bk7**	Rice	*Pseudomonas fuscovaginae*	Antibiosis	*In vitro, in greenhouse*	93% Inhibition rate; 76.6%	(Ullah Kakar et al., 2014)
***B. subtilis* PTS-394**	Pepper	*Fusarium solani*	Antibiosis (CLPs) and ISR	*In vitro, in greenhouse* and *in field*	69,63% Biocontrol efficacy; 74,43% Biocontrol efficacy	(Qiao et al., 2023)
***B. subtilis* 168**		*Ralstonia solanacearum*	ISR (VOCs)	*In vitro*	ns	(Yi et al., 2016)
***Bacillus subtilis* HSY21**	Soybean	*Fusarium oxysporum*	ns	*In vitro, in greenhouse* and *in field*	81.30% Inhibition rate; 63.83% control effects; 57.07% control effects	(Han S. et al., 2021)
***B. amyloliquefaciens* Q-426**	Spinach	*Fusarium oxysporum* f.sp *spinaciae*	Antibiosis (CLPs)	*In vitro*	Approx. 28 mm inhibition zone diameter	(Zhao et al., 2014)
***B. subtilis* CAS15**	Sweet pepper	*Fusarium oxysporum* f.sp *capsici*	Competition for nutrients and ISR	*In greenhouse*	Reduction of the disease incidence by 12.5 to 56.9%	(Yu et al., 2011)
***B. megaterium* TRS-4**	Tea	*Fomes lamaoensis, Sphaerostilbe repens, Poria hypobrumea, Sclerotium rolfsii*	Antibiosis and ISR	*In vitro, in greenhouse*	55–84% Inhibition rate	(Chakraborty et al., 2006)
***B. amyloliquefaciens* XJ5**	Tomato	*Alternaria solani*	Antibiosis (CLPs, lytic enzymes)	*In vitro*	82.5% Inhibition rate	(Mu et al., 2023)
***B. velezensis* K01**		*Botrytis cinerea*	Antibiosis (CLPs, lytic enzymes)	*In vitro, in vivo* (on detached leaves and harvested fruits)	84.1% Inhibition rate, >80% Disease reduction, >78% Disease reduction	(Xue et al., 2023)
***B. subtilis* EPC016**		*Fusarium oxysporum* f.sp. *lycopersici*	Antibiosis (VOCs, lytic enzymes), Competition for nutrients (siderophore)	*In vitro, in greenhouse*	46.04% to 60.78% Inhibition rate	(Ramyabharathi and Raguchander, 2014)
***B. velezensis* UQ9000N**		*Fusarium oxysporum* f.sp. *lycopersici*, *Macrophomina phaseolina*	Antibiosis	*In vitro*	52% to 56% inhibition rate	(Arkhipov et al., 2023)
***B. velezensis* FJAT-46737**		*Ralstonia solanacearum*	Antibiosis	*In vitro, in greenhouse*	Approx. 82.0% Biocontrol efficiency	(Chen et al., 2020))
***Bacillus amyloliquefaciens* FJAT-2349**			Antibiosis (CLPs)	*In greenhouse*	97.6% Biocontrol efficiency	(Chen M. et al., 2019)
***B. amyloliquefaciens* PKM16**		*Sclerotinia sclerotiorum*	Antibiosis	*In vitro, in greenhouse*	40.27% Inhibition rate	(Do Prado Mattos et al., 2023)
***B. subtilis* IJ10**	Turmeric	*Fusarium solani, Pythium aphanidermatum*	Antibiosis (VOCs, lytic enzymes)	*In vitro, in greenhouse*	>45% Inhibition rate; >60% Biocontrol efficacy	(Kharshandi and Kayang, 2023)

ns, not specified.

In addition to their efficacy against phytopathogenic fungi and bacteria, some *Bacillus* spp. have been reported for their potential to control nematodes, insects and weeds (Mnif and Ghribi, 2015). For instance, surfactins from *B. velezensis* S499 showed insecticidal activity against the fruit fly *Drosophila melanogaster* (Assié et al., 2002). The wheat rhizosphere *Bacillus altitudinis* D30202 was shown to have bioherbicidal potential on the grass *Avena fatua* L. (Ma et al., 2023). *Bacillus* strains such as *B. altitudinis*, *B. pumilis*, *B. velezensis*, *B. mojavensis* and *B. megaterium* showed nematicidal activity against *Meloidogyne incognita* (Padgham and Sikora, 2007; Huang et al., 2010; Xiang et al., 2017; El-Nagdi and Abd-El-Khair, 2019; Guimarães Pacifico et al., 2021; Ye et al., 2022) and *Heterodera glycines*, which threatens soybean production (Dalvan Do Nascimento et al., 2022). Cell-free culture supernatants and volatiles of *B. amyloliquefaciens* BV03, PTA4838 and *B. velezensis* MBI600 killed more than 85% of *Helicotylenchus dihystera* infesting soybean (Camatti et al., 2023). So even if very few products have been developed to the market scale so far, the results already available are promising for the design of bionematicides, bioinsecticides and bioherbicides based on *Bacillus*.

## 9 Conclusions

This review summarizes the current phytosanitary situation in the GLCCA region and the possibility of shifting to safer and more environmentally friendly alternatives to chemical pesticides for sustainable agriculture. Several diseases and pests are devastating many crops, leading to the use of chemical pesticides despite their known hazardous nature. Although pesticide regulations exist in the three countries, their implementation is problematic with one third of registered pesticides already banned from market in EU and numerous mishandling incidents. For the sake of the terrestrial and aquatic ecosystems, governments in the GLCCA need to take urgent action to mitigate the harmful effects of chemical pesticides. The regulatory systems should be reviewed and updated, and other less toxic pesticides or more sustainable control strategies should be promoted. Inspectors should be increased in number and motivation, and retailers and farmers should be made aware of pesticide regulations and the associated health and environmental risks.

Globally available alternatives inspired by integrated pest management (IPM), including good agricultural practices and biological control, are not widely used and/or in some cases non-existent. Although a limited number of biocontrol products are registered in the region, their use has yet to become a “reality”. Moreover, all available products are used against insect pests but there is no biological solution that has been developed in practical terms to fight highly prevalent fungal and bacterial diseases. Some *Bacillus* isolates clearly represent promising tools for the control of these microbial diseases, and their inherent ability to form resistant endospores, to grow on poor substrates such as agro-industrial residues in semi-solid systems, and their potential to secrete a vast array of bioactive secondary metabolites (Kumar et al., 2021; Bouassida et al., 2023) would allow designing stable, rich, and affordable formulations of products suitable for the local socio-economic context. Unfortunately, although some preliminary studies have demonstrated efficacy in controlling some important local pathogens, these *Bacillus*-based products are still a “myth” for local farmers. Efforts should be directed toward promoting the use of already available *Bacillus*-based products against endemic pathogens devastating crops in local farming systems. However, bioprospecting for indigenous *Bacillus* strains that are well-adapted to local agro-ecological conditions and retain strong biocontrol properties should be promoted to ensure sustainable plant disease management in the region. The great potential of indigenous *Bacillus* strains over the commercial ones has been shown, for example in the management of grapevine trunk disease (Langa-Lomba et al., 2023) and tropical fruit diseases (Reyes-Estebanez et al., 2020). Governments of the GLCCA countries should not only encourage the investment in such biocontrol products, which are virtually absent in the region, but also facilitate their registration and commercialization.

## Author contributions

GN: Conceptualization, Writing – original draft. VKA: Conceptualization, Writing – original draft. FN: Conceptualization, Writing – original draft. VN: Funding acquisition, Writing – review & editing. AL: Conceptualization, Supervision, Writing – review & editing. MO: Conceptualization, Funding acquisition, Project administration, Supervision, Writing – review & editing.
