# Supplementary material for: Bacillus-based biocontrol beyond chemical control in central Africa: the challenge of turning myth into reality
Source: Front Plant Sci. 2024 Feb 6;15:1349357. doi: 10.3389/fpls.2024.1349357 (PMC10877027; doi:10.3389/fpls.2024.1349357)
Supplement: Supplementary file 1 [file Table_1.docx]

Supplementary Material

***Bacillus*-based biocontrol beyond chemical control in central Africa: the challenge of turning myth into reality.**

Gaspard Nihorimbere^1,6*†^, Virginie Korangi Alleluya^2,4*†^, François Nimbeshaho^2,5*†^, Venant Nihorimbere^3^, Anne Legrève^1*^ and Marc Ongena^2*^

**Correspondence:**Gaspard Nihorimbere [gaspard.nihorimbere@uclouvain.be](mailto:gaspard.nihorimbere@uclouvain.be); [nihorimbereg@yahoo.fr](mailto:nihorimbereg@yahoo.fr);

Virginie Korangi Alleluya : [v.korangi@doct.uliege.be](mailto:v.korangi@doct.uliege.be), [virginiekorangi@gmail.com](mailto:virginiekorangi@gmail.com);

François Nimbeshaho : [fnimbeshaho@uliege.be](mailto:fnimbeshaho@uliege.be); [francois.nimbeshaho@gmail.com](mailto:francois.nimbeshaho@gmail.com);

Anne Legrève [anne.legreve@uclouvain.be](mailto:anne.legreve@uclouvain.be);

Marc Ongena [marc.ongena@uliege.be](mailto:marc.ongena@uliege.be)

# Table S1: Registered pesticides in the GLCCA countries. R, registered; NR, not registered; Names in red, EU banned pesticides.

| Class of pesticides | Active ingredients | Burundi | DRC | Rwanda |
| --- | --- | --- | --- | --- |
| Insecticides + Acaricides | Abamectin | R^[[1]](#footnote-1)^ | R | NR^[[2]](#footnote-2)^ |
|  | Acephate^[[3]](#footnote-3)^ | R | NR | NR |
|  | Acetamiprid | R | R | R |
|  | Acrinathrin | NR | NR | R |
|  | Alpha-cypermethrin | R | NR | R |
|  | Aluminium phosphide | R | NR | R |
|  | Azocyclotin | NR | NR | R |
|  | Benfuracarb | R | NR | NR |
|  | Beta-cyfluthrin | R | NR | R |
|  | Bifenthrin | R | R | R |
|  | Bromopropylate | NR | NR | R |
|  | Buprofenzin | NR | NR | R |
|  | Carbofuran | NR | NR | R |
|  | Carbosulfan | NR | NR | R |
|  | Chlorfenapyr | NR | NR | R |
|  | Chlorpyriphos | R | NR | NR |
|  | Chlorpyriphos-ethyl | R | R | NR |
|  | Chlorpyriphos-methyl | R | NR | NR |
|  | Clofentezin | NR | NR | R |
|  | Copper oxychloride | NR | R | NR |
|  | Cyfluthrin | R | NR | NR |
|  | Cypermethrin | R | R | R |
|  | Cyphenothrin | NR | R | NR |
|  | Cyromazine | NR | NR | R |
|  | Deltamethrin | R | R | R |
|  | Diafenthiuron | NR | NR | R |
|  | Diazinon | R | NR | NR |
|  | Dichlorvos | R | NR | NR |
|  | Diflubenzuron | NR | NR | R |
|  | Dimethoate | R | R | NR |
|  | Esfenvalerate | R | R | NR |
|  | Fenamiphos | NR | NR | R |
|  | Fenaxaquin | NR | NR | R |
|  | Fenbutatin oxide | NR | NR | R |
|  | Fenitrothion | R | R | NR |
|  | Fenthion | R | NR | NR |
|  | Fenvalerate | R | NR | NR |
|  | Fipronil | R | R | R |
|  | Flufenoxuron | NR | NR | R |
|  | Flumethrin | NR | NR | R |
|  | Imidacloprid | R | R | R |
|  | Indoxacarb | NR | NR | R |
|  | Isoxathion | R | NR | NR |
|  | Lambda-cyhalothrin | R | R | R |
|  | Lufenuron | R | NR | NR |
|  | Magnesium Phosphide | R | NR | R |
|  | Malathion | R | NR | R |
|  | Methomyl | NR | NR | R |
|  | Methoxyfenozide | NR | NR | R |
|  | Nicosulfuron | NR | R | NR |
|  | Novaluron | NR | NR | R |
|  | Omethoate | R | NR | NR |
|  | Oxydemeton-methyl | R | NR | NR |
|  | Permethrin | NR | NR | R |
|  | Piperonyl Butoxide (PBO) | NR | NR | R |
|  | Pirimiphos | R | NR | R |
|  | Pirimiphos-Methyl | NR | NR | R |
|  | Profenofos | NR | NR | R |
|  | Prosuler Oxamatrine | R | NR | NR |
|  | Pymetrozine | R | NR | NR |
|  | Pyridaphenthion | NR | R | NR |
|  | Pyrimicarb | R | R | R |
|  | Snake repellent | NR | NR | R |
|  | Spiromesifen | NR | NR | R |
|  | Tau-fluvalinat | NR | NR | R |
|  | Teflubenzuron | NR | NR | R |
|  | Tetradifon | NR | NR | R |
|  | Tetramethrin | NR | R | NR |
|  | Thiacloprid | NR | NR | R |
|  | Thiamethoxam | NR | R | R |
|  | Tralomethrin | R | NR | NR |
|  | Triazophos | R | NR | NR |
|  | Zeta-cypermethrin | R | NR | NR |
| Fungicides | Albesilate | NR | NR | R |
|  | Azoxystrobin | R | NR | R |
|  | Benalaxyl | NR | NR | R |
|  | Benomyl | R | NR | R |
|  | Bitertanol | NR | NR | R |
|  | bromoxinyl-octanoate | NR | NR | R |
|  | Bupimate | NR | NR | R |
|  | Calcium Hypochlorite | NR | NR | R |
|  | Captan | NR | NR | R |
|  | Carbendazim | NR | NR | R |
|  | Carboxin | NR | NR | R |
|  | Chlorothalonil | R | NR | R |
|  | Copper | NR | NR | R |
|  | Copper ammonium acetate | NR | NR | R |
|  | Copper hydroxide | R | R | R |
|  | Copper oxychloride | R | R | R |
|  | Cuprous Oxide | NR | NR | R |
|  | Cymoxanil | NR | NR | R |
|  | Cyproconazole | NR | NR | R |
|  | Dichlofluanid | NR | NR | R |
|  | Didecyldimethylammonium chloride | NR | NR | R |
|  | Difenoconazole | NR | R | R |
|  | Dimethomorphe | NR | NR | R |
|  | Dithianon | NR | NR | R |
|  | Dodemorph acetate | NR | NR | R |
|  | Epoxiconazole | NR | NR | R |
|  | Fenarimol | NR | NR | R |
|  | Fenamidone | NR | NR | R |
|  | Fenhexamid | NR | NR | R |
|  | Flutriafol | NR | NR | R |
|  | Fluzilazol | NR | NR | R |
|  | Fosetyl - Aluminium | R | NR | R |
|  | Hexaconazole | NR | R | R |
|  | Imidachloriprid | NR | NR | R |
|  | Iprobenfos | R | NR | R |
|  | Iprodione | R | NR | R |
|  | Iprovalicarb | NR | NR | R |
|  | Isoxadifen-ethyl | NR | NR | R |
|  | Kresoxim-methyl | NR | NR | R |
|  | Mancozeb | R | R | R |
|  | Maneb | R | NR | NR |
|  | Mefenaxam | NR | NR | R |
|  | Metalaxyl | R | R | R |
|  | Metiram | NR | NR | R |
|  | Micronised Sulphur | NR | NR | R |
|  | Penconazole | NR | NR | R |
|  | Pencycuron | NR | NR | R |
|  | Prochloraz | R | NR | NR |
|  | Propamocarb hydrochloride | NR | NR | R |
|  | Propineb | NR | NR | R |
|  | Pyrimethanil | NR | NR | R |
|  | Spiroxamine | NR | NR | R |
|  | Sulphur | NR | R | R |
|  | Tebuconazole | R | NR | R |
|  | Thiabendazole | NR | NR | R |
|  | Thiophanate methyl | R | R | R |
|  | Thiram | R | R | R |
|  | Tolclofos Methyl | NR | NR | R |
|  | Triadimefon | NR | R | NR |
|  | Triadimenol | NR | R | NR |
|  | Tricyclazole | NR | NR | R |
|  | Trifloxystrobin | NR | NR | R |
|  | Triforine | NR | NR | R |
|  | Vinchlozoline | NR | NR | R |
| Herbicides | 2,4-D | R | NR | R |
|  | 2,4-DB | R | NR | NR |
|  | Ametryn | R | NR | NR |
|  | Atrazine | R | R | NR |
|  | Bispyribac sodium | NR | R | NR |
|  | Clethodim | NR | NR | R |
|  | Dalapon | R | NR | R |
|  | Dimethamethryn | R | NR | NR |
|  | Diuron | R | NR | R |
|  | Fluazifop butyl | NR | R | NR |
|  | Fluazifop-p-butyl | R | NR | NR |
|  | Fluroxypyr | NR | R | NR |
|  | Fluroxypyr ester-methylheptyl | NR | R | NR |
|  | Glyphosate | R | R | R |
|  | Hexazinone | R | NR | NR |
|  | Isoproturon | R | NR | NR |
|  | Linuron | NR | NR | R |
|  | Methribuzin | NR | NR | R |
|  | Metolachlor | R | NR | R |
|  | Metribuzin | R | NR | NR |
|  | MSMA | R | NR | NR |
|  | Nicosulfuron | NR | R | NR |
|  | Oxadiazon | NR | R | NR |
|  | Oxyfluorfen | NR | NR | R |
|  | Penoxsulam | NR | R | NR |
|  | Promethrin | R | NR | NR |
|  | Propanil | NR | NR | R |
|  | Tembrotrione | NR | NR | R |
|  | Terbutryn | NR | NR | R |
|  | Thiobencarb | NR | NR | R |
|  | Triclopyr | NR | R | NR |
| Rodenticides | Brodifacoum | R | NR | R |
|  | Bromadiolone | R | R | R |
|  | Chlorophacinone | R | NR | NR |
|  | Coumatetralyl | R | NR | R |
|  | Difenacoum | R | NR | R |
|  | Diphacinone | R | NR | R |
|  | Flocoumafen | R | NR | NR |
| Nematicides | Dazomet | R | NR | R |
|  | Fenamiphos | NR | NR | R |
|  | Oxamyl | NR | R | NR |
|  | Terbufos | R | NR | NR |
| Molluscicides | Mercaptodimethur | NR | NR | R |
|  | Metaldehyde | R | NR | R |
| Growth regulators | Serricornine | R | NR | NR |
|  | Etherphon | NR | R | NR |
|  | Daminozide | NR | NR | R |
| Biopesticides | Azadirachtin | NR | NR | R |
|  | *Bacillus thuringiensis* | R | R | R |
|  | *Beauveria bassiana* | NR | NR | R |
|  | Pyrethrins | NR | NR | R |
|  | Spinosad | NR | NR | R |
|  | *Trichoderma harzianum* | NR | NR | R |
|  | Emamectine benzoate | NR | R | NR |

1. [↑](#footnote-ref-1)
2. [↑](#footnote-ref-2)
3. [↑](#footnote-ref-3)
